# Real-Time Secure/Unsecure Video Latency Measurement/Analysis with FPGA-Based Bump-in-the-Wire Security

**DOI:** 10.3390/s19132984

**Published:** 2019-07-06

**Authors:** Admir Kaknjo, Muzaffar Rao, Edin Omerdic, Thomas Newe, Daniel Toal

**Affiliations:** Department of Electronic and Computer Engineering, Centre for Robotics and Intelligent Systems (CRIS) University of Limerick, V94 Limerick, Ireland

**Keywords:** AES, IoT, video, IoVT, marine, FPGA, LabVIEW FPGA, RoV, IoRT

## Abstract

With the growth of the internet of things (IoT), many challenges like information security and privacy, interoperability/standard, and regulatory and legal issues are arising. This work focused on the information security issue, which is one of the primary challenges faced by connected systems that needs to be resolved without impairing system behaviour. Information, which is made available on the Internet by the things, varies from insensitive information (e.g., readings from outdoor temperature sensors) to extremely sensitive information (e.g., video stream from a camera) and needs to be secured over the Internet. Things which utilise cameras as a source of information pertain to a subclass of the IoT called IoVT (internet of video things). This paper presents secured and unsecured video latency measurement results over the Internet for a marine ROV (remotely operated vehicle). A LabVIEW field programmable gate arrays (FPGAs)-based bump-in-the-wire (BITW) secure core is used to provide an AES (advanced encryption standard)-enabled security feature on the video stream of an IoVT node (ROV equipped with a live-feed camera). The designed LabVIEW-based software architecture provides an option to enable/disable the AES encryption for the video transmission. The latency effects of embedding encryption on the stream with real-time constraints are measured and presented. It is found that the encryption mechanism used does not greatly influence the video feedback performance of the observed IoVT node, which is critical for real-time secure video communication for ROV remote control and piloting. The video latency measurement results are taken using 128, 256 and 512 bytes block lengths of AES for both H.264 and MJPEG encoding schemes transmitted over both TCP and UDP transmission protocols. The latency measurement is performed in two scenarios (i.e., with matching equipment and different equipment on either end of the transmission).

## 1. Introduction

The number of things that are connected to the Internet is growing considerably. Because of this, a new concept, called IoT (internet of things) is emerging [1]. The IoT is a developing research area that has technical, social and economical significance. Technical significance results because IoT potentially enables us to connect, manage and control devices on the Internet, which were previously unconnected. The social significance is because interconnection of so many things will result in a multi interconnected smart world, in which many of the machines and the elements around us will be communicating with each other without any kind of human intervention. This certainly has the potential to improve the quality of human life. Lastly, the economic significance of the IoT is because it could be the largest driver of economic growth and employment in the next decade. The IoT is further divided into subclasses like; IoVT (internet of video things), IoRT (internet of remote things), IoV (internet of vehicle) [2], IIoT (industrial internet of things) etc. The work reported in this paper comes under the IoT subclass of IoVT and IoRT [3,4].

Things equipped with a camera sensor stand out from other things due to the potentially sensitive nature of information and high bandwidth requirements for the video stream transfer. Thus, a subclass of IoT called IoVT was introduced, which addresses interconnection of things with cameras as sensors [5]. While the IoRT addresses things distributed over the wide geographical area [6], which in many cases do not have a terrestrial link to the Internet, but have to rely on satellite communication instead.

Lack of implemented security on interconnected devices makes the IoT a darker world of surveillance; because in the IoT environment everything is potentially accessible through the Internet. So, ensuring information security on the Internet is very important to build trust in the use of Internet enabled devices. If people believe that their connected devices and their information on the Internet are not secure from misuse or harm, then they will be reluctant to use these devices. On the other hand, it is crucial that enabling information security does not significantly alter performance of the overall IoT systems.

As security in the IoT is an important aspect, ranging from the technological issues to the more philosophical ones, such as trust and privacy, the security threats in the IoT can be divided into two categories [7]: physical attacks and non-physical attacks. The physical attacks are related to the physical nature of the things that are usually deployed in the public areas and cannot be constantly supervised like: cloning of things, malicious substitution of things, firmware replacement attack and extraction of security parameters. The non-physical attacks are related to the threats when the things start communication with each other like: eavesdropping attack, man-in-the-middle attack, routing attacks and denial of services. Protection from eavesdropping attack is in focus of this paper.

In recent years, devices with integrated micro controllers, referred to as “things” in the IoT lexicon, are being transformed from simple and low-power devices to complex devices which often utilise power-hungry sensors (e.g., cameras) and possess complex hardware architectures (e.g., based on field programmable gate arrays; FPGAs). The main advantage of FPGA platforms as compared to ASIC (application specific integrated circuit) platforms is flexibility, which makes an FPGA a popular platform in IoT [8]. This makes the FPGA easy to interface to, for example, temperature, pressure, position, acceleration, ADC and DAC converters, etc., which is of great importance to IoT devices since these interfaces are frequently required by the “things”. Another reason for using the FPGA in IoT is that the IoT devices have long life spans. Manufacturers may stop developing and rolling out software patches for a product once it reaches obsolescence. This is a reason why security provision should likely be implemented with reconfigurable hardware (such as FPGAs) in IoT rather than with software patches.

The FPGAs (field programmable gate arrays) platform is very suitable for the implementation of cryptographic algorithms, because of its re-configurability and high performance features [9]. Initially, the IoT was specifically linked with resource-constrained environments but. today, the IoT has become a popular term, which is largely accepted to include all mobile and fixed devices (including the FPGA; i.e., modern reconfigurable hardware platforms) with Internet connectivity and computing capability [10,11].

Although this research is conducted by utilizing marine robot application, the methods and results presented here can be beneficial for any remote control systems with video feedback. The encryption method is designed as a portable solution, which can be easily applied to various remote control systems. The analysis of latency (both video and network) is of great importance for systems with real-time constraints. The impact of latency on control systems is highlighted in [12,13]. Here, the technical, experimental and procedure details involved to develop the system are explained in detail to make reproducibility of the work easy for researchers in the field.

Main contributions of this work are as follow:

- An improved version of our previously published work [12] in terms of having support for encrypted video on demand, while all previous functionalities are preserved.
- To the best of author(s) knowledge, this is the first work which provides secure and unsecure video latency measurement detail analysis and comparison.
- The implemented encryption does not have noticeable effect on video quality.
- This is the first work which presents the video latency measurement results using two scenarios based on same hardware and different hardware on each end of the video communication. This shows the adoptability of the proposed work for different platforms.

The remainder of the paper is organized as follow: Section 2 provides problem definition, Section 3 provides related literature review, Section 4 covers the details of the proposed video latency measurement technique, Section 5 describes the test configurations, Section 6 provides performance results, Section 7 contains discussion and comparison of results and Section 8 concludes.

## 2. Problem Definition

The importance of data security in IoT applications is highlighted in the introduction section. Data security is important for practically every Internet-connected device. The complexity of data security implementation tasks varies greatly and depends on the application and on the industry requirement for which the device is used. For example, implementation of data security for distributed control systems with real-time constraints is considered a highly challenging task. In such systems, real-time constraints apply on the process of data security provision/implementation. Distributed systems, which are connected through various networks and/or over the Internet are susceptible to latencies in data transfer, hence data processing, such as security implementation, must be conducted within the shortest possible time in order not to impair the functionality of the system. Latency analysis is an important factor in real-time communication. The developed system measures/analyses the secure video communication latency using a number of configurations based on different hardware, encoding schemes, block sizes of encryption algorithm and transport protocols.

A marine system, which pertains to IoVT and IoRT, used for IRM (inspection, repair and maintenance) of wind farms and other remote offshore structures, is used as the target application in this work. This system is developed by the CRIS (Centre for Robotics and Intelligent Systems) at the University of Limerick [13]. This developed system consists of a marine ROV, which is controllable remotely over the Internet and a control station. All features of the robotic system (i.e., an on-board camera, connectivity over the Internet and the lack of a security mechanism) made it a perfect platform for experiments conducted for the purpose of this work.

**Figure 1:**
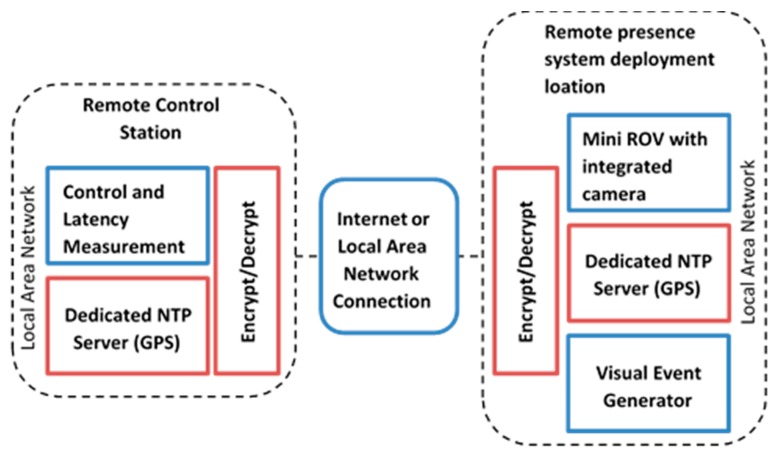
Target application system with integrated encryption and latency measurement system.

The work of this paper mainly involves secure and unsecure video latency measurement over the Internet. As mentioned above, the target application is marine, which involves the remote control of an ROV from a control station. The encryption level security is implemented using a previously published AES [14] algorithm implementation on FPGA [15,16], where AES is implemented on a traditional FPGA and then imported into LabVIEW FPGA to use with a developed LabVIEW application for video latency measurement. The AES security feature is controlled using the enable/disable option in the LabVIEW software. The structure of the target application is shown in Figure 1. It depicts the remote control station and the robot deployment location as two main components of the system. The two locations can be connected either through a LAN or through the Internet. On both ends there are dedicated stratum 1 NTP servers which are used as the source for precise clock synchronization. Two main applications are also shown (i.e., a visual event generator that is used for generating events, which are captured by the camera and a latency measurement application (used for calculating glass-to-glass latency). The video stream is passed through the LabVIEW application, which encrypts the data. The encryption is conducted on encoded video data. Similarly, before the decoding process takes place on the receiving end, the stream has to pass through the decryption process. The encryption and decryption process is done by the same application, and that is why the application is named as Encrypt/Decrypt.

As mentioned previously, in this work an FPGA is used to provide AES security and the comparison of traditional FPGA and LabVIEW FPGA is given in [14].

## 3. Literature Review

This section includes a literature review of the following: video latency measurement, packet switch network, time sensitive networking and secure video.

Video latency measurement: This work is based on our previously published work [12] which focused on the measurement of glass-to-glass video delays [17]. In Ref. [12], a comprehensive review of state-of-the-art techniques for measuring latency of the video were covered. Here, some techniques and tools of video latency measurement are listed and a short description is provided.

The first tool is called AvCloak [18] and is intended to be used mostly in conference call applications. The tool is able to provide OWD (one-way delay) measurements [19], with two ends synchronised to generic NTP servers. A tool with similar technique and target application is called vDelay [20]. Both tools focus on capturing timestamps as part of video data on one end and decoding of the information for calculating the difference on the opposite end. The VideoLat [21] tool measures the RTD (round-trip delay) of the video. This tool does not require any form of time synchronization while timestamp information is embedded into the video signal in the form of QR codes. Authors in Ref. [22] investigate the effects of video latency in augmented reality applications while in Ref. [23] novel latency reduction algorithm are presented. In these two papers, an LED was used as the source for the visual event.

It is worth noting that none of the cited papers investigated video latency measurement with and without encryption. In addition, there is no detailed analysis of the network latency component within the glass-to-glass video latency, which becomes particularly interesting if the video is transferred over a packet-switched network, which is the case for this paper.

Packet switch network: A characteristic of packet-switched networks is that they do not guarantee delivery of packets within a bounded time. The end-to-end delay of a network packet consists of the following delays: processing, egress and ingress delay [24], packets queuing delay [25] and transmission and propagation delays [26]. Successful packet delivery depends on the transport layer protocols (TCP or UDP) used. The TCP protocol guarantees that all packets will be delivered to the destination. There is a possibility that some packets will be delayed and retransmitted several times before the acknowledgement receipt by the receiving end [27]. On the other hand, the UDP protocol does not guarantee the packet delivery, but it also does not negatively impact end-to-end delays [28]. All mentioned delay factors make transfer of the video in real-time over a network a challenging task.

The evaluation metrics for the QoS (quality of service) in real-time is mandatory for certain applications like controlling a robot based on the video feedback over the Internet/LAN, which is the target application of this paper. The capabilities of data transfer speeds on Ethernet, excellent electromagnetic compatibility [29] and low cabling costs of Ethernet are encouraging factors for this application. The main goal is to enable guaranteed transfer of video data in real-time.

Time sensitive networking: In recent years huge effort has been put into development of deterministic communication capabilities between devices connected over standard Ethernet. The most valuable progress, which resulted in a set of networking standards, was made by the TSN (time sensitive networking) task group which is a part of the IEEE 802.1 working group. The TSN was formed from the AVB (audio video bridging) task group which was dealing with real-time transport of audio and video over Ethernet [30]. The resulting solutions are defined by four main IEEE standards: 802.1AS, 802.1Qat, 802.1Qav and 802.1BA [31]. Guaranteed delivery with minimum bounded latency, low jitter and packet loss are the main goals or achievements aimed for by the TSN standards [32]. The main idea behind the TSN mechanisms is splitting the network traffic into three types based on priorities (listed in descending order): TT (time-triggered traffic), AVB traffic and BE (best-effort) traffic [33]. New capabilities have enabled usage of Ethernet in different industries (e.g., automotive, automation and aviation) for communication in processes which have hard real-time requirements for video transfer. A sample application where TSN was utilised is presented in [34]. Guaranteed end-to-end latency of 2 ms over seven hops is the main characteristic of this TSN network [35]. Despite performance improvements introduced by time sensitive networking when compared with standard Ethernet, the main area of application of the technology is in industrial local area networks (e.g., real-time systems in a car, plane, etc.). It is also worth noting that in order to establish a time sensitive network, it is necessary to use dedicated network devices, which support this technology. This is due to the fact that the technology is implemented on layer 2 (the MAC layer) of the OSI model [36].

Secure video: Adding encryption to the video signal in order to provide the data security makes real-time video transfer more complex. Encryption of the video stream data has been an active field of research for a long time. The IoVT technology increased the number of video streaming devices on the Internet, which in turn increases the necessity for having high quality security solutions. Existing video encryption solutions differ based on the amount of video stream data that actually gets encrypted. On this basis, there are partial and full video encryptions. With partial techniques, encryption is conducted on part of the data necessary for scrambling the video and making it worthless without decrypting. A full video encryption technique implies encryption of the complete content of the video stream, making it more secure, but at the same time the process of full encryption requires more processing power. Full encryption can be realized by using two techniques called homomorphic and invariant encryption [37]. These two differ by the amount of signal processing, which can be conducted on the encrypted video signal and also differ by efficiency. The first technique is more efficient while the latter one offers more flexibility in terms of signal processing. The ability to process and alter encrypted signals is important in cases where new information needs to be embedded into the stream (e.g., watermark embedding). Depending on the application of encrypted video, both full and partial encryption techniques have drawbacks and benefits. The main drawbacks for full video stream encryption include increased video latency, high required processing power and inability to process encrypted video without decrypting it. For this reason, a lot of effort has been made in algorithms, which can be used for partial encryption of video streams. An algorithm which deals with selective encryption for H264 video streams is published in Ref. [38], while in [39,40] selective encryption mechanism are proposed to be used mainly with the HEVC (high efficiency video coding) compression standard. In contrast to selective video encryption, work which deals with full encryption of video streams were also published [41]. In Ref. [42], authors propose an algorithm which solves a three-fold goal in video encryption research: high compression, low computation cost and secrecy. The authors use an FPGA platform for implementation of the algorithm and focus on ensuring real-time video delivery.

In Ref. [43], authors implemented encryption of real-time video stream by using several different encryption algorithms. Asymmetric key cryptographic transform based on elliptic curve cryptography (ECC) and AES methods were used. The encryption was implemented for the purpose of testing and analysing the performance of different ECC curves, which were then compared with the performance of AES-encrypted and non-encrypted video streams in streaming applications. Real-time is defined as continuous flow of the media stream over the Internet where the content is presented before it has been downloaded completely. End-to-end delay, jitter, packet loss, encryption and decryption times and data rates were measured and the results were analysed for a total of 18 tested curves and for the case with no encryption. All tests were conducted on an institutional LAN (local area network) and by using MJPEG for encoding the video. The RTSP (real-time streaming protocol) [44] was used for transferring the video and full encryption of the video stream data was conducted. The presented end-to-end delay values do not exceed 10 ms for any curve. The encryption time of AES was found to be in sub-millisecond range. It is worth noting that in the paper there are no details about the technique used for delay measurement.

The encryption technique presented in Ref. [45] was applied to video stream data and transferred over the Internet using software designed to support video and network latency measurement. The stream was encrypted by using the efficient high speed AES algorithm, which is considered to be the most suitable for encryption of the real-time video in terms of speed and frame size overhead [46]. The algorithm is implemented on a LabVIEW FPGA platform [14]. Implementation of the algorithm on hardware is mandatory for real-time performance of the encryption, especially in the case of full video stream encryption.

This work provides a detailed technique for secure video transmission with thorough analysis of latency. Video and network latencies were simultaneously measured by using the software application designed/presented in this paper. While the tools presented in [18,20,21] provide a means for video latency measurement, none of the listed papers explicitly deal with encrypted video. Use of time sensitive networks represent an interesting approach, especially because the packet delivery is guaranteed and delivery takes place within bounded time. However, they can only be applied on video applications, which are running inside controlled environments (LAN in a car or in industrial applications). Since communication over the Internet is the focus of this paper, the usage of time sensitive networks becomes unfeasible. This paper deals with fully encrypted video streams, which represent a more secure option when compared with partially encrypted video. For this reason, dedicated FPGA platforms were used. End-to-end latency for various elliptic curves in real-time encryption and streaming applications was measured and analysed in [43], but these tests were conducted within an isolated LAN. In addition, no video latency was measured and analysed.

Publications which are most relevant to this work are listed in Table 1. The classification was made based on the following criteria: Network type determines whether the papers deal with LAN or Internet; time-sync provides information about the way of synchronising time; latency type, which was measured and presented, is listed in column “Latency”; information about the encryption algorithm used is given in the “Encryption” column and the column titled “Latency” lists best achieved results. The only comparable results, to those presented in this section, are the ones which were published in our previous work [12].

## 4. Proposed Video Latency Measurement Technique

The video latency measurement solution, which was developed and presented in [12], was upgraded here by adding the possibility for full encryption of the video stream data. The best available way to add security services is using hardware (FPGA)-based implementation, as the software-based implementation introduced significant delays into the video data transmission. The solution presented here can be applied to any system, which features video transfer over the Internet (or a LAN) in real-time.

The proposed solution is highly portable and can be used for latency measurements of unsecure/secure video streams in any system which possesses transfer of video over IP. In addition, the proposed solution is modular and can be easily adapted to implement higher levels of security, instead of only encryption security.

The proposed architecture is based on the video server component described in [12], which was upgraded with applications, which are able to run on platforms with NI Linux RTOS (real-time operating system) [47]. In addition, the modules which perform the encryption/decryption and have to be executed on the FPGA platform were also integrated into the software of [12]. The major applications were developed in LabVIEW, while video decoding was performed by a third party component called FFmpeg [48] (version 4.0 built by gcc (GNU Compiler Collection) 7.3.0).

**Figure 2:**
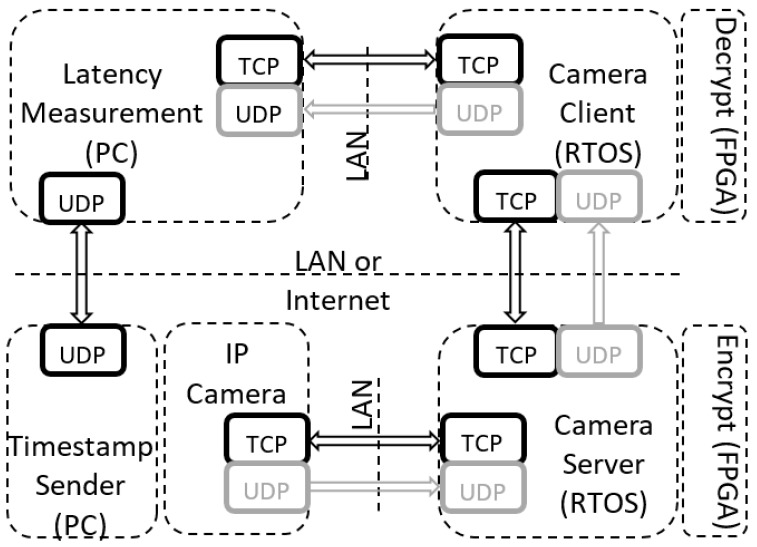
Software architecture.

As shown in Figure 2, the architecture consists of the following nodes:

- Timestamp Sender
- Camera Server
- Encrypt/Decrypt Applications
- Camera Client
- Latency Measurement.

**Figure 3:**
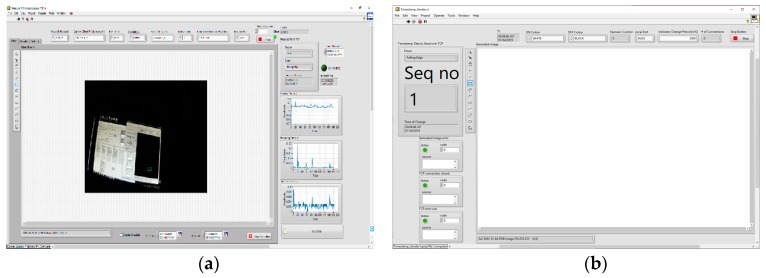
Screenshots of applications, (**a**) latency measurement and (**b**) timestamp sender.

Timestamp sender and latency measurement applications screenshots of Figure 2 are presented in Figure 3. The latency measurement application is the starting point for running of all tests (all other applications must be in running mode before executing this application). The purpose of this application is to initiate communication with other applications, which should result in receiving the IP camera video stream. Other applications from Figure 2 serve as clients/servers for parsing the data, encrypting/decrypting the stream when necessary and for providing reference time for latency measurement (timestamp sender). The main functionalities of the latency measurement application are: decode video and measure video latency. The IP camera node in Figure 2 represents a physical device with its own firmware. The rest of the nodes represent LabVIEW applications running on either: Windows PCs, NI Linux RTOS or FPGA targets. Connections marked with LAN denote links over Gigabit Ethernet network. All nodes of Figure 2 exchange information by using UDP and TCP protocols.

This architecture can be used in different configurations using options of protocol type, encryption string length and encoding algorithm. In terms of protocol type, there are two possible choices: UDP and TCP. This option modifies the way video data is streamed from the IP camera to the latency measurement node. When UDP is selected, all channels (both TCP and UDP) shown in Figure 2 are used. In this case, all video stream data are transferred over the UDP while TCP is used only for sending RTSP protocol packets. When TCP is selected all data is transferred over TCP connection only, greyed out UDP channels in Figure 2 are not used in this case. The encryption string length denotes the maximum amount of data which can be encrypted/decrypted at once. For this we used three sets of strings; 128 bytes, 256 bytes and 512 bytes. Additionally, there are two options available to select the encoding algorithm (i.e., MJPEG or H264). Encoding of the data was done by the IP camera, while the stream was decoded in the latency measurement application utilizing the FFMPEG library.

Details of each node are given below:

### 4.1. Timestamp Sender

**Figure 4:**
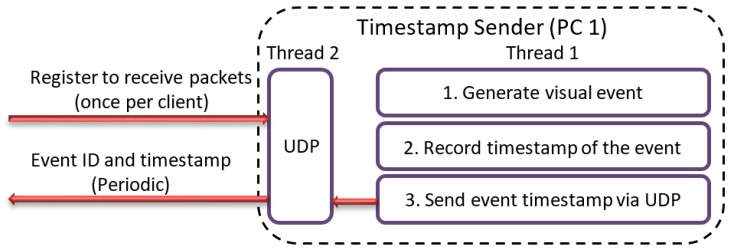
Timestamp sender application.

Timestamp Sender is used for generating a visual event, which is captured by the camera’s sensor. Each event is accompanied with the data containing the timestamp of the event and event ID. The event ID is a number which is incremented for each generated event. This number is displayed in the application window which is captured by the IP camera. The purpose of the event ID is to indicate the quality of the video for the end user. The timestamp is captured from windows system time with 1 ms resolution. The application acts as a server in a way that keeps a pre-defined UDP port open for client registration. Any client that sends a request packet to the port will be registered and will keep receiving the visual event data periodically. The described functionality is shown in Figure 4.

### 4.2. Camera Server

This application node runs on the real-time target and is responsible for communication with the camera, preparing the data for encryption, adding timestamp information to packets and sending encrypted data back to the client. The server accepts connections on a pre-defined TCP port which is used for the RTSP transport initialization procedure [44]. RTSP packets are accepted, parsed and forwarded to the IP camera, while responses from the IP camera are handled in a similar way and are returned to the client, which initiated the RTSP connection in the first place. The following protocol methods are implemented: OPTIONS, DESCRIBE, GET_PARAMETER, SETUP, PLAY and TEARDOWN.

Once the connection parameters are negotiated, the video data stream is started by sending the PLAY command from the application. It is worth noting that video data are encoded by either H264 or MJPEG encoding algorithms and this process is done by the IP camera before the transmission of the data to server application. Depending on the desired transport protocol for video data transfer (TCP or UDP), the stream can be transferred over the same TCP connection on which RTSP was initiated or it can be transferred over a UDP port, which needs to be negotiated in the RTSP initialization procedure.

**Figure 5:**
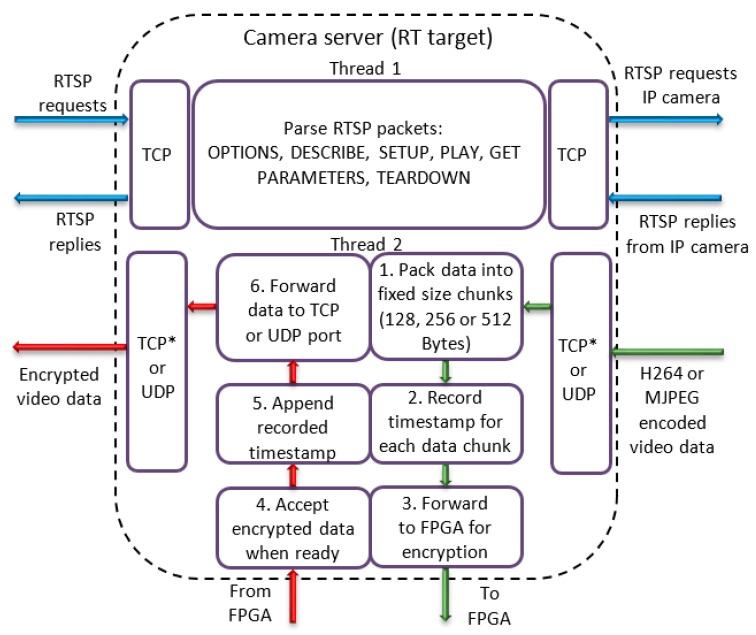
Camera server application.

The application node is organized in two threads as can be seen in Figure 5. One thread is responsible for parsing the RTSP protocol while another thread is used for manipulation of video data. Once the stream commences, the data packets sent from the camera are accepted by the application and are reorganized into chunks of 128, 256 or 512 Bytes (Step 1), depending on data length encryption settings. At this point, a timestamp is taken from the real-time operating system (Step 2) for appending to the data after encryption is finished (Step 5). Fixed size chunks are then transferred to the FPGA application node which is responsible for encrypting the data (Step 3). When encryption is finished, data is read from the FPGA (Step 4) and forwarded to the TCP or UDP port (Step 6).

### 4.3. Encrypt/Decrypt Applications

These application nodes run on FPGA targets and are responsible for encryption and decryption of data. The BITW (bump-in-the-wire) security solution, which is explained in [45], has been applied to video data for the purpose of this work. The efficient AES encryption algorithm implementation is used for encryption/decryption of the data. The AES was implemented in two phases [14]. In the first phase, AES was designed for a standard FPGA platform using Verilog and in the second phase the traditional FPGA implementation was imported to LabVIEW FPGA using the IPIN node.

Data transfer between applications running on the real-time processor (camera client and camera server) and applications running on the FPGA (encrypt/decrypt) is based on writing data to controls and reading data from indicators created in the FPGA application. For this purpose, a block called read/write control is used by the software on the real-time processor. For each control and indicator, a fixed size register is created on the FPGA to be accessed by the real-time processor. The main advantage of this type of data transfer, when compared to other methods [49,50], is low overhead. Additionally, with each call of the read/write control function, data transfer is initiated with minimum delay. The main disadvantage is in the fact that data transfer is not based on DMA (direct memory access) and, hence, requires the real-time processor to be available to handle it.

**Figure 6:**
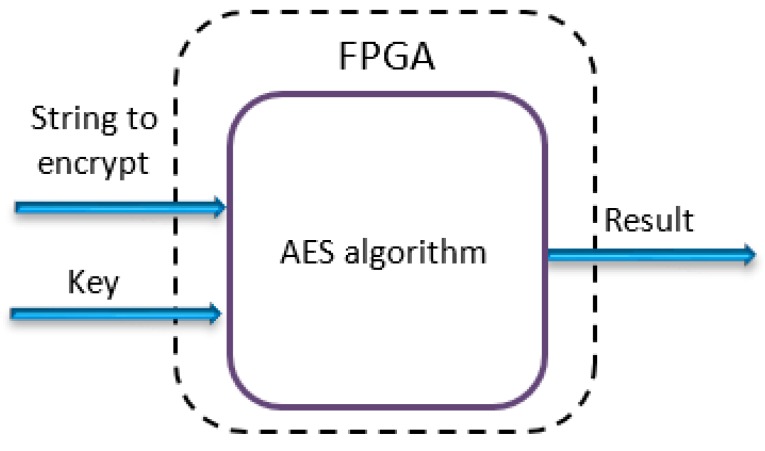
Field programmable gate array (FPGA) node for encryption/decryption of the data.

In order to decrypt data, it is necessary that both encrypt and decrypt VIs have the same 128-bit key set. Figure 6 shows the block diagram of the FPGA application node. Once the data is encrypted/decrypted, the result (which has the same size as the input data) is written to the output register of the FPGA target and is read by the application (camera server or camera client) running on the real-time processor.

### 4.4. Camera Client

**Figure 7:**
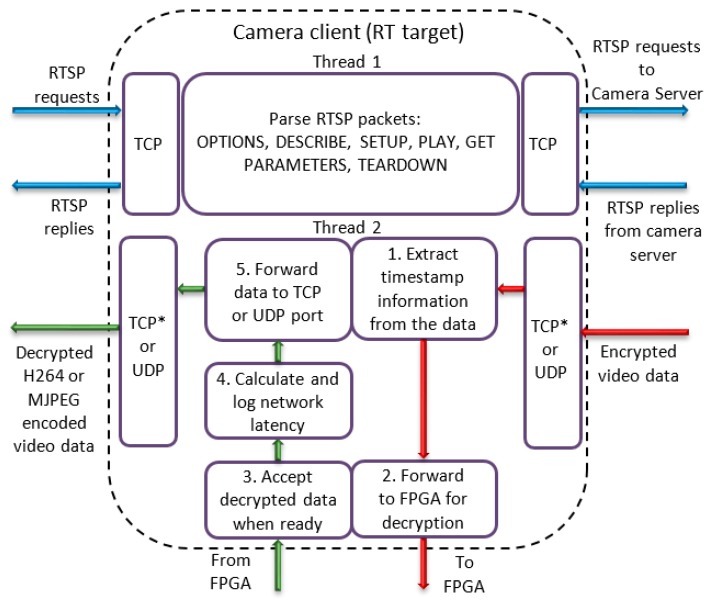
Camera Client application.

This application node, which is also running on the real-time target, initiates connection and receives video data from the camera server application. The functionality related to parsing the RTSP packets is the same as in the server application. The client application listens on a pre-defined TCP port for incoming connections, which will be propagated to the IP camera over the camera server application. Similarly, the video stream data, which is decrypted in the client application, is sent back to the latency measurement application. Like the camera server application, the camera client application is also organized in two threads, as shown in Figure 7. The first thread handles the RTSP protocol, while the second one is responsible for parsing the video data.

The first step after receiving a fixed size data packet from the server is to the extract timestamp information from the packet (Step 1). After that, the packet is forwarded to the FPGA application node, which performs data decryption (Step 2). When the FPGA finishes decryption and decrypted data is read by the client application (Step 3), the network latency is calculated by subtracting the timestamp, captured from the real-time operating system time after decryption is completed (Step 4) from the timestamp, which arrived together with the data packet (extracted in Step 1). The calculated network latency is logged to file on the real-time target (Step 4). In Step 5, the decrypted video data is forwarded to the main application. It is worth noting that the data after decryption is again encoded with H264 or MJPEG algorithms (depending on the video source encoding type).

### 4.5. Latency Measurement

This application node is responsible for various important functionalities, which include connection to the camera client, the starting sequence of RTSP packets, accepting and decoding video data, detecting events in the video and calculating the video latency. The latency measurement node also registers to the timestamp sender application node in order to be able to receive event ID and timestamp data, based on which the video latency is calculated. A pre-built FFmpeg library was used for handling the RTSP and decoding of the video. The image processing and event detection is done by utilizing the NI vision development module.

**Figure 8:**
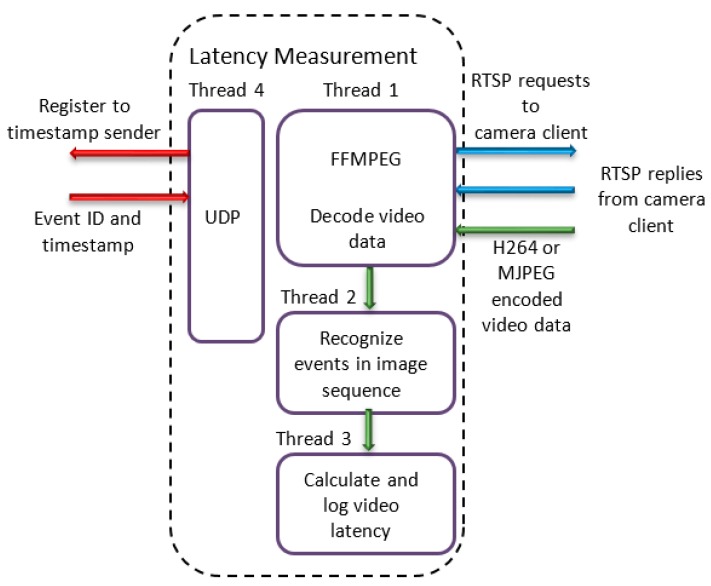
Latency measurement application architecture.

As illustrated in Figure 8, the application is organised in four threads. The first thread utilises a pre-built FFMPEG library for starting the RTSP sequence and for decoding the video data. The output of Thread 1 is a sequence of images, which are fed to Thread 2. Data transfer between the threads is done by using queues in order to ensure that no data will be lost during the transfer. Thread 2 applies the IMAQ colour learn [51] function from the NI vision development module to each image in the sequence. The function outputs an image colour spectrum which contains the colour features present in the image. Whenever a visual event (which was generated by the timestamp sender application) is detected, the associated timestamp is captured and Thread 3 is notified. This thread is responsible for calculating the video latency, which is done by subtracting the timestamp at the moment the visual event was detected from the timestamp at the moment the same event was generated. The thread also logs the calculated latency data to a file. Times needed for the image to be processed by the application (overhead time) is also taken into account and subtracted from the final result. Thread 4 is responsible for registering to the timestamp sender application and receiving visual event data from it. The data that is received by Thread 4 is forwarded to Thread 3 so that the necessary calculation can be done.

## 5. Test Configurations

**Figure 9:**
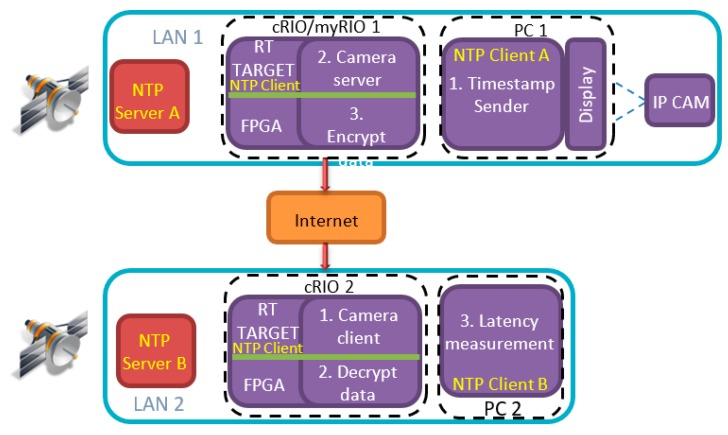
Video latency measurement with encryption over the Internet.

The proposed architecture presented in Section 4 can be employed with all components within a LAN or it can be used over the Internet. A configuration of the measurement system, when components are connected over the Internet is shown in Figure 9. The components inside each LAN are connected by Gigabit Ethernet. As well as PCs, an IP camera and NTP servers, there are also two devices which feature on-board real-time processor and FPGA chips. The devices are of type cRIO-9034 from National Instruments with Intel Atom E3845 CPU running the 64-bit NI Linux real-time operating system. The on-board FPGA chip is of type Xilinx Kintex-7 7K325T and is connected to the CPU by PCI bus. As Figure 9 shows, nodes for data encryption and decryption run on the FPGA, while the camera server and camera client nodes run on the real-time processor.

IP camera model Hikvision 2CD2010F-I was used in all tests, with the following encoding parameters: resolution (640 × 480), bitrate type (variable), video quality (lowest), frame rate (25) and max bitrate (2048). As mentioned earlier, two options for encoding algorithms are possible: H264 and MJPEG. Video encoding is done on the transmitting end Dell inspiron 15 5000 series laptop PC with an intel core i7-8550U @ 1.80 GHZ processor and Intel UHD Graphics 6.20, while video decoding is done on the receiving end of the test setup by using a Dell Inspiron 7720 laptop PC with an Intel core i7-3630QM @ 2.4 GHz Processor and an NVIDIA GeForce GT 650 M graphics card.

During all the tests, IP camera was located at the University of Limerick, Ireland with Internet connection bandwidth of 100 Mbps for both upload and download. In cases when myRIO was used for decryption, it was located in Sarajevo (Bosnia and Herzegovina) with Internet connection bandwidth of 4 Mbps for download and 2 Mbps for upload. In another case, when both cRIO devices are located in Limerick, Ireland, receiving end of test setup was connected to the Internet with bandwidth 96.2 Mbps for download and 86.0 Mbps for upload.

**Figure 10:**
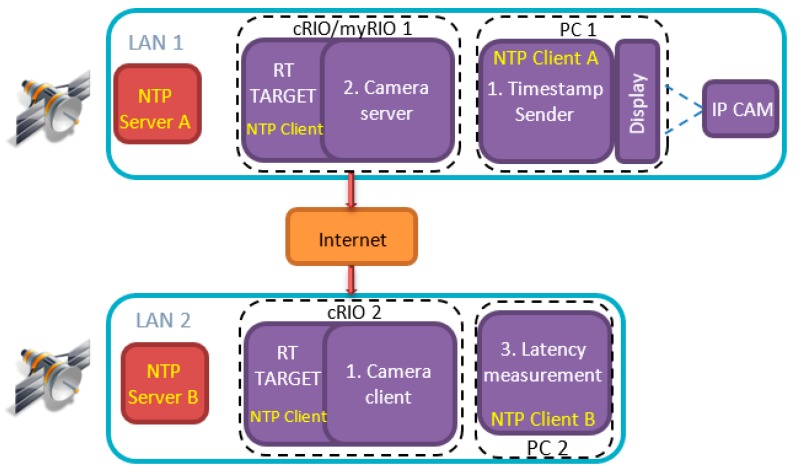
Video latency measurement without encryption over the Internet.

The configuration shown in Figure 10 was used for the case when no encryption is used. In comparison to Figure 9 the only difference in Figure 10 is the absence of encrypt/decrypt applications, which run on the FPGA target. The data is still routed through cRIO devices, but in this case, there is no transfer to the FPGA and processing of the data.

The flow of data in the experimental configuration with encryption is as follows:

(1) The timestamp sender application (PC1) generates periodic visual events.
(2) The camera server (cRIO 1) opens an RTSP [44] connection to the IP camera.
(3) The camera client (cRIO 2) opens an RTSP connection to the camera server.
(4) The latency measurement application (PC2) opens a connection to the camera client.
(5) The camera server generates a timestamp before data encryption.
(6) The video data is encrypted on the FPGA and the data is streamed to the listening application.
(7) The camera client receives and unpacks each TCP/UDP frame.
(8) The FPGA decrypts the video data.
(9) The camera client calculates the network delay for each packet.
(10) The latency measurement application (PC2) receives visual event information from timestamp sender.
(11) When an event is detected, video latency is calculated and stored.

It is also worth noting that encryption/decryption functionality can be easily enabled or disabled by changing the state of a switch in the software. In addition, if there is a need to test the performance of different encryption algorithms, the test can be modified in a way that only the code on FPGA is replaced, while the rest of the system remains the same.

Here, the test configurations are designed as “offline” tests of video and network latency. It means the system being tested, cannot do its normal functions until the test is completed. The reason for this is that there has to be a dedicated PC with display to run the timestamp sender application. However, if there is a need to have information about video latency “online,” while the system is in normal operation, it can be done by slightly modifying the hardware and software used. Such software modification includes a change in visual event generator, which would have to be designed in a way that it is permanently installed into the camera’s field of view. In hardware modification, an embedded system with precision timing and LEDs would have to be considered instead of a PC with a display and timestamp sender application. The described modifications are not implemented as part of this publication, but are suggestions for future work.

## 6. Performance Results

This section provides details of performance results. Each combination of options, which is supported by the equipment, was tested. These include the following options: encoding algorithm, transport protocol, encryption on or off and encrypted string length. The motivation for such comprehensive testing lies in the desire to achieve the following goals: draw conclusion about the effect of encryption on video and network latencies; determine a set of parameters which result in optimal performance of encrypted video in terms of video latency; compare the latencies of encrypted and unencrypted video streams; compare obtained results to our previously published work [12]. For the purpose of neat result presentation, the tests are divided based on different scenarios, groups and cases.

Scenario based on hardware platform: This scenario is based on a hardware platform, used for running camera client/camera server and encrypt/decrypt applications. This scenario is called symmetrical (Scenario I) for the case when the same equipment was used on both ends of the test setup and called non-symmetrical for the case when different equipment was used on both ends (Scenario II). Detailed results are provided for the symmetrical scenario only, while for the non-symmetrical scenario, the main conclusion and figures are shown.

Groups based on encryption string length: Each scenario is further divided into groups based on encryption string length (Group 1/Group 2/Group 3 correspond to 128/256/512 bytes, respectively).

Cases based on encoding algorithm and transport protocol: Each group consists of four cases which were derived by using different combinations of options for the encoding algorithm (H264/MJPEG) and the transport protocol (TCP/UDP). For each case, a pair of tests were conducted, one without encryption and the other with encryption. For each test, two sets of results were obtained. One set represents video latency, while the other set represents network latency. The configuration without encryption is shown in Figure 10. All tests were conducted with equipment connected over the Internet.

Two scenarios (symmetrical and non-symmetrical) were necessary in order to show the portability of the developed system to different hardware platforms. The total number of cases in both scenarios was 20, of which 12 cases pertain to scenario I and 8 cases to scenario II. The total amount of tests and presented data may seem large, but this amount of detail is necessary in order to explore all possible options and the measurements supported by the proposed system. This thorough analysis helps in the conclusions drawn about the best approach.

In the symmetrical scenario, two compactRIO devices were used as hardware platforms to run the camera client/camera server together with the encrypt/decrypt application nodes. Here, three groups of tests based on string length (128/256/512 bytes) were used. Both ends of the testing setup were located in Limerick, Ireland for the tests.

### 6.1. Video Latency

**Figure 11:**
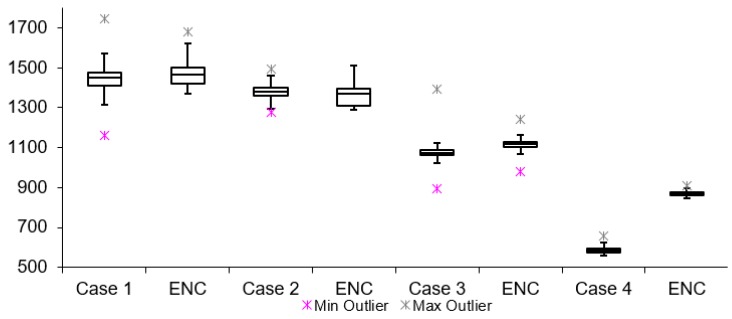
Video latency Group 1.

**Figure 12:**
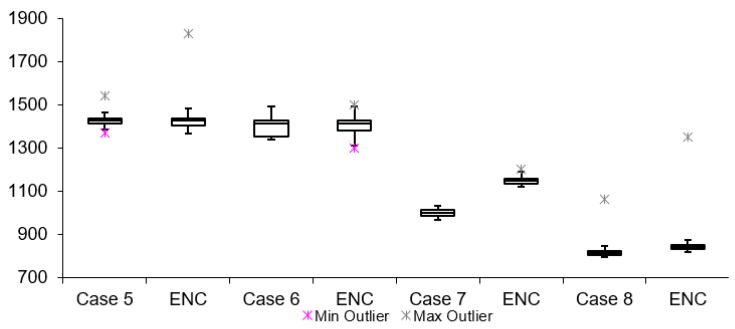
Video latency Group 2.

**Figure 13:**
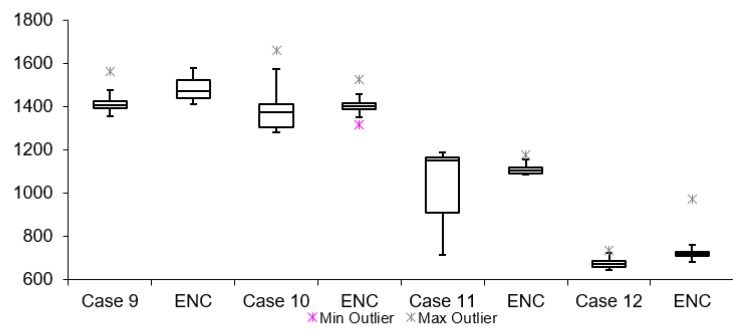
Video latency Group 3.

Table 2 lists twelve cases of video latency results. For each combination of transport protocols (TCP/UDP) and encoding types (H264/MJPEG), a test with and without encryption was conducted. When comparing arithmetic mean values, it is noticeable that for all cases, tests with encryption resulted in overall higher latency. This is expected due to the fact that there is additional data processing in tests with encryption. The difference varies from 3 to 300 ms for different cases. The results for Group 1, 2 and 3 are presented in Figure 11, Figure 12 and Figure 13, respectively. Measurement results are presented in the form of box plots. Tests without encryption are denoted with case number while the same tests with encryption are denoted with ENC.

Comparison can be made based on the mean video latency measurements for secure and insecure video. In most cases, tests with encryption have higher video latency values than the tests without encryption. There are two cases which can be considered the best: Case 5 and Case 6. The difference between encrypted and unencrypted video latency is 1.76 ms (in Case 5) and 3.08 ms (in Case 6). In addition, it is found that when encryption is enabled, cases with MJPEG encoding algorithms perform worse than cases with H264. This can be attributed to the nature of the encoding algorithms, i.e., the amount of data which needs to be encrypted and transferred in MJPEG compared to H264. The worst option to use when encryption is enabled is Case 4. This conclusion is made based on the difference in video latency values of tests conducted with and without encryption.

### 6.2. Network and Encryption Latency

**Figure 14:**
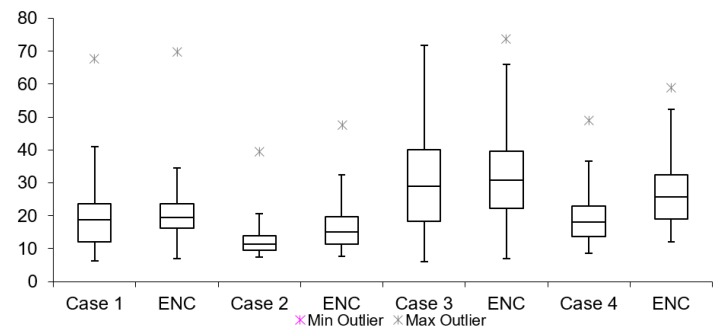
Internet network latency (128 bytes encryption).

**Figure 15:**
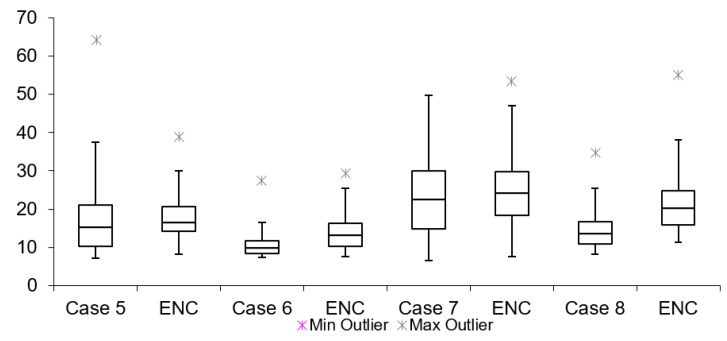
Internet network latency (256 bytes encryption).

**Figure 16:**
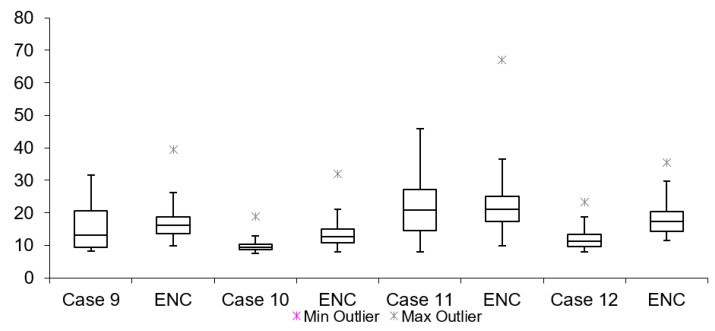
Internet network latency (512 bytes encryption).

Table 3 lists network latency results for all cases mentioned in Table 2. The network and encryption latency is defined as the time which passes from the time when the TCP/UDP packet starts in the encryption process to the time when decryption of the same packet is finished on the receiving end of the setup. These values are logged on real-time devices used during the tests. Table 3 shows that all tests without encryption have lower latencies when compared to tests with encryption. The maximum difference is lower than 1 ms (Case 11). This proves that having encryption of the data does not have a large influence to overall video latency. Summarized results for Group s1, 2 and 3 are shown in Figure 14, Figure 15 and Figure 16, respectively.

By looking at the network latency information, it is possible to get a deeper insight into the causes of the overall latency of the video. Network latency itself results from several factors: packet processing, egress and ingress delays, packets queuing delay and transmission and propagation delays. However, the measurements presented here correspond to network latency of the packets together with the time taken for the encryption/decryption process. Similarly, for video latency measurements in this scenario, all cases have a mean latency value lower in tests without encryption than in tests with encryption.

In this scenario, for each transport protocol and encoding algorithm, six tests were conducted: three tests with unencrypted video streams and three tests using encryption. The difference between the three tests was in the encrypted string length (128, 256 and 512 bytes). Although it was not found that the video latency in cases with 128 and 256 bytes string lengths performed worse than the video latency in cases with 512 bytes string length, the option with 512 bytes string length is preferred because of lower count of encryption/decryption calls. Having larger chunks of data is beneficial because of the issue known as the “small-packet problem” for UDP. The TCP protocol implements Nagle’s algorithm which deals with the problem by buffering packets and sending several small packets as one [52].

**Figure 17:**
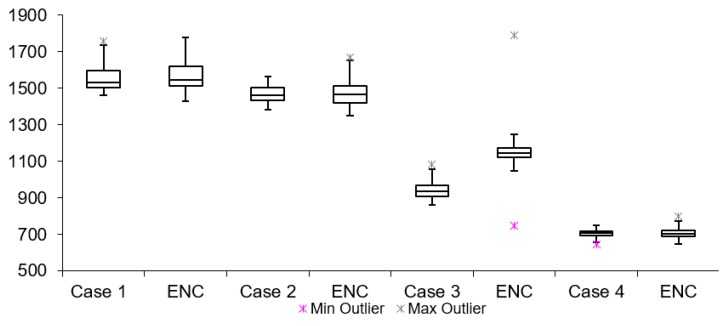
Scenario I video latencies.

**Figure 18:**
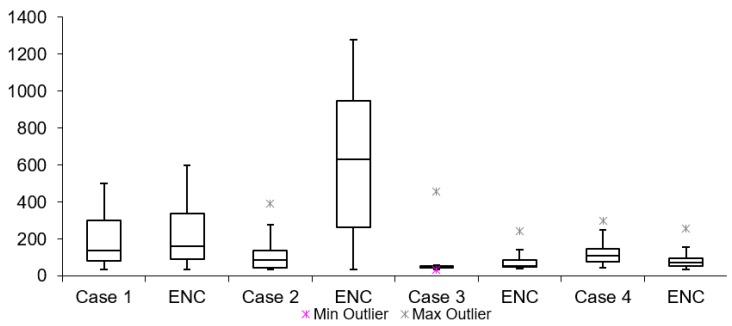
Scenario I network latency.

The equipment configuration, shown in Figure 9, is used for testing in the non-symmetrical scenario. Here, the camera client and decrypt application nodes run on a NI myRIO, while the camera server and encrypt application nodes run on a NI cRIO (compactRIO) device, and only a 128 byte string length is used due to the limits of the FPGA chip area in the NI myRIO. It is worth noting that part of the setup used in this scenario was located in Limerick (IP camera and cRIO), while the other part was located in Sarajevo (myRIO). Video latency measurement results are shown in Figure 17, while network latency measurements are shown in Figure 18. By looking at Figure 17, it is noticeable that in all cases, except in Case 4, tests with encryption do have higher mean values when compared to the tests without encryption. This is expected due to the fact that there is additional data processing in tests with encryption. However, Case 4 stands out with the same resulting arithmetic mean latency in both tests, with and without encryption. It can happen that other causes of video latency overpower and cancel the delays introduced by the additional calculations needed in cases with encryption. This conclusion is based on results presented in Figure 18, where it is visible that network latency with secured video is higher than the latency with an unsecure video stream. This case proves that the security solution, which was added in the loop, does not significantly influence the latency of the video, since the variation in network latency can be higher than delays introduced by the encryption process.

## 7. Discussion

In this paper, full encryption of the video stream was conducted. The stream was encrypted in order to evaluate the security solution in terms of video and network latency and to test its usability in applications, which demand real-time transfer of the video over the Internet.

The literature review section has highlighted the latest achievements in several fields, which are related to topics discussed in this paper such as: video latency, video security and latency of secured video as the most important. It is worth noting that no results which could be directly compared to ours were discovered in the literature. The results which were closest to ours were from tests in a LAN, with only network latency measurements. In order to compare the results, the secured video would need to be transferred over the Internet and have the video latency measured.

As mentioned earlier, the lowest video latency obtained here was in Case 4 (without encryption) and it had the value of 586.16 ms. The lowest secure video latency measured was in Case 12 (i.e., 724.86 ms). The lowest value of latency for secure and unsecure video were obtained using the combination of MJPEG and UDP. These two cases differ in the length of the encrypted data string. Case 4 pertains to 128 bytes string length and Case 12 is conducted with encryption string length of 512 bytes.

When comparing the results presented in this paper to the results published in Ref. [12], it is noticeable that relations between cases with different options are kept the same in this paper. H264 encoding still results in greater video latency than MJPEG. In terms of the underlying protocol for transporting the video, an observation can be made that cases with the TCP protocol result in higher latency values than cases with UDP. In terms of absolute latency values, it is noticeable that results presented in this paper had generally higher values than the ones published previously. This is because of the existence of two additional nodes in the network between the ends of the test setup. Despite the fact that these devices operate in real-time, the transfer of data to and from the devices takes time, which accounts for additional video latency. The BITW technique and encryption algorithm itself does not have a significant influence on the latency values, but having two extra nodes in the network certainly does cause increased delays.

To further compare the results, it is worth noting that results obtained over the Internet have been taken into consideration. In Ref. [12], the minimum video latency was 558.05 ms for the case when the UDP protocol was used to transfer video encoded with the MJPEG algorithm. In this work, the minimum video latency was also achieved in the same case (i.e., UDP protocol and MJPEG encoding) and this measured value was 586.16 ms. The increase of 28.11 ms in the mean video latency value is attributed to the existence of the two additional nodes between video source and destination. Additionally, for all other cases in this paper, the mean video latency was higher when compared to previous results of Ref. [12] with equivalent tests.

Results obtained by using UDP as the transport protocol had lower increase in video latency when compared to results obtained with TCP. The reason for this lies in the difference in UDP and TCP protocol implementations. The UDP protocol does not guarantee the packets delivery and in-order delivery. TCP protocol ensures the guaranteed and in-order delivery. Having all parts of the video stream in the right order is crucial for the video quality. One of the main causes of increased latency with TCP is retransmission. This retransmission occurs whenever a packet or a packet segment is not acknowledged by the destination node. Retransmission happens after a timeout known as RTO (retransmission timeout).

When compared to tests without encryption, it was found that introducing AES encryption does not necessarily or significantly increase video latency. This is because of the FPGA-based high speed implementation of the AES core using a BITW approach. The LabVIEW–FPGA-based implementation of 128 bytes and 256 bytes AES was achieved at 200 MHz, while 512 bytes AES implementation was achieved at 160 MHz and throughput achieved for all mentioned block sizes was in the range of Gbps [15]. Adding two additional nodes near the camera and a node on the receiving end of the stream, proved to have a greater influence on the latency values than the encryption process itself. Based on the presented results with encryption, it is clear that using H264 encoding over TCP outperforms the other cases in terms of video latency.

## 8. Conclusions

Full encryption of video streams with real-time requirements was conducted for a remote-control marine-based application with live video feedback to a human pilot. Network and video latencies were measured for different video encoding algorithms and encryption string size settings. In terms of video stream, H.264 and MJPEG encoding algorithms were tested, while the video stream was encrypted with string sizes of 128, 256 and 512 bytes.

In order to perform full encryption of the video in real-time, two FPGA-based devices were added to the system. Network traffic (containing a video stream) was routed through these nodes and encrypted/decrypted. It is visible from the presented results that enabling encryption using the BITW technique with AES counter mode (AES-CTR) does not greatly influence performance of the video stream in terms of latency. Although in most tests the latencies were marginally higher with encryption, there were a few cases where it was not the case, which indicates that there are other factors (related to the camera and its output stream) which potentially have greater influence on the latency than the encryption itself. The benefits gained by having an encrypted video stream are numerous (privacy and data protection) and outweigh the costs (latency) of the encryption itself.

To avoid or reduce this latency, one of the targets for future work is to integrate the security directly into the camera (i.e., at the video source) and also into the equipment on the receiving end of the stream. However, this approach nullifies the major advantage of realising provision of end-to-end security using a bump-in-the-wire technique where the BITW device can be retrofitted to legacy infrastructure that does not have security capabilities. This BITW retrofitting provides a cost-effective solution for security upgrading of expensive equipment.

## Figures and Tables

**Table 1:** Summary of literature review techniques and results.

Tool/Work	Network	Time Sync	Latency	Encryption	Latency [ms]
Our previous work [12]	Internet	Dedicated NTP	Network	N/A	40.92
	LAN	Dedicated NTP	Network	N/A	1.52
	Internet	Dedicated NTP	Video	N/A	558.00
	LAN	Dedicated NTP	Video	N/A	488.00
TSN [35]	LAN	IEEE 1588-2008	Video	N/A	<2.00
Sen [43]	LAN	N/A	Network	AES, ECC	approx. >4.0
Bachhuber [17,23]	N/A	N/A	Video	N/A	19.18
Jacobs [22]	N/A	N/A	Video	N/A	40.00
vDelay [20]	Internet	Generic NTP	Video	N/A	69.00
AvCloak [18]	Internet	Generic NTP	Video	N/A	50.00
VideoLat [21]	Internet	N/A	Video	N/A	N/A

**Table 2:** Scenario I test video latency results.

Test Name		Protocol	Encoding	Encrypted	Data Length	Arithmetic Mean [ms]	Median [ms]	Min Value [ms]	Max Value [ms]	Standard Deviation [ms]
Group 1	Case 1	TCP	H264	NO	128	1449.01	1448.36	1163.24	17422.85	52.57
		TCP	H264	YES	128	1468.00	1464.37	1368.38	1680.23	55.37
	Case 2	UDP	H264	NO	128	1376.72	1379.02	1276.35	1493.63	33.01
		UDP	H264	YES	128	1382.22	1387.32	1341.13	1474.67	26.04
	Case 3	TCP	MJPEG	NO	128	1071.16	1071.52	894.56	1392.23	37.29
		TCP	MJPEG	YES	128	1117.00	1117.66	979.33	1239.15	21.15
	Case 4	UDP	MJPEG	NO	128	586.16	584.14	557.27	656.12	14.62
		UDP	MJPEG	YES	128	868.33	867.08	845.42	910.08	11.89
Group 2	Case 5	TCP	H264	NO	256	1429.15	1428.59	1368.62	1541.27	24.82
		TCP	H264	YES	256	1430.91	1426.33	1365.82	1828.51	44.35
	Case 6	UDP	H264	NO	256	1399.67	1393.27	1293.2	1522.47	34.87
		UDP	H264	YES	256	1402.75	1414.91	1299.60	1499.92	40.47
	Case 7	TCP	MJPEG	NO	256	998.38	999.15.	964.13	1031.71	16.22
		TCP	MJPEG	YES	256	1150.59	1147.76	1121.58	1202.80	14.46
	Case 8	UDP	MJPEG	NO	256	816.02	816.08	794.52	876.98	12.31
		UDP	MJPEG	YES	256	843.19	843.74	820.94	883.43	11.17
Group 3	Case 9	TCP	H264	NO	512	1413.37	1409.64	1355.42	1562.79	33.75
		TCP	H264	YES	512	1481.81	1469.33	1412.66	1578.85	45.33
	Case 10	UDP	H264	NO	512	1372.62	1375.25	1282.46	1660.71	66.47
		UDP	H264	YES	512	1411.26	1408.40	1386.23	1543.13	21.98
	Case 11	TCP	MJPEG	NO	512	1047.59	1150.67	713.31	1191.16	128.47
		TCP	MJPEG	YES	512	1108.69	1106.81	1086.19	1179.45	16.62
	Case 12	UDP	MJPEG	NO	512	673.76	671.40	647.55	736.3	15.62
		UDP	MJPEG	YES	512	724.86	720.32	682.40	974.49	39.81

**Table 3:** Scenario II test network latency results.

Test Name		Protocol	Encoding	Encrypted	Data Length	Arithmetic Mean [ms]	Median [ms]	Min Value [ms]	Max Value [ms]	Standard Deviation [ms]
Group 1	Case 1	TCP	H264	No	128	19.38	18.74	6.19	67.64	9.16
		TCP	H264	Yes	128	20.68	19.57	6.94	69.84	6.99
	Case 2	UDP	H264	No	128	12.56	11.36	7.54	39.52	4.45
		UDP	H264	Yes	128	16.41	15.05	7.71	47.42	6.26
	Case 3	TCP	MJPEG	No	128	29.73	29.07	6.18	71.86	13.54
		TCP	MJPEG	Yes	128	31.45	30.73	6.94	73.68	11.47
	Case 4	UDP	MJPEG	No	128	18.80	18.08	8.73	48.98	6.34
		UDP	MJPEG	Yes	128	26.22	25.74	12.00	58.99	8.58
Group 2	Case 5	TCP	H264	No	256	16.30	15.33	7.27	64.00	7.17
		TCP	H264	Yes	256	16.93	16.60	8.13	38.92	5.23
	Case 6	UDP	H264	No	256	10.56	9.76	7.43	27.51	2.77
		UDP	H264	Yes	256	13.92	13.19	7.70	29.36	4.28
	Case 7	TCP	MJPEG	No	256	22.81	22.49	6.63	49.69	9.38
		TCP	MJPEG	Yes	256	24.37	24.09	7.65	53.13	7.47
	Case 8	UDP	MJPEG	No	256	14.37	13.64	8.23	34.78	4.33
		UDP	MJPEG	Yes	256	20.92	20.32	11.27	54.92	6.24
Group 3	Case 9	TCP	H264	No	512	14.78	13.05	8.20	31.56	5.72
		TCP	H264	Yes	512	16.37	16.24	9.92	39.44	4.59
	Case 10	UDP	H264	No	512	9.73	9.35	7.49	18.97	1.64
		UDP	H264	Yes	512	13.14	12.62	8.04	31.95	3.14
	Case 11	TCP	MJPEG	No	512	20.88	20.75	8.06	45.96	7.57
		TCP	MJPEG	Yes	512	21.36	21.04	9.78	67.08	5.60
	Case 12	UDP	MJPEG	No	512	11.74	11.13	7.99	23.21	2.75
		UDP	MJPEG	Yes	512	17.73	17.33	11.52	35.39	4.07
